# Red Cell Death in Renal Disease: The Role of Eryptosis in CKD and Dialysis Patients

**DOI:** 10.3390/cells14130967

**Published:** 2025-06-24

**Authors:** Grazia Maria Virzì, Anna Clementi, Claudio Ronco, Monica Zanella

**Affiliations:** 1 Department of Nephrology, Dialysis and Transplant, St Bortolo Hospital, 36100 Vicenza, Italy; cronco@goldnet.it (C.R.); monica.zanella@aulss8.veneto.it (M.Z.); 2 IRRIV—International Renal Research Institute Vicenza, 36100 Vicenza, Italy; a.clementi81@virgilio.it; 3 Department of Nephrology and Dialysis, Santa Marta and Santa Venera Hospital, 95024 Acireale, Italy

**Keywords:** eryptosis, CKD, red blood cells, dialysis

## Abstract

Eryptosis is a programmed cellular death involving red blood cells (RBCs). It is a physiological mechanism that leads to the removal of defective erythrocytes, similarly to apoptosis. Its typical features are cell shrinkage, cell membrane blebbing, and membrane scrambling with the consequent exposure of the aminophospholipid phosphatidylserine on the outer surface of RBCs. Different mechanisms play a role in the pathogenesis of eryptosis, such as the increase in cytosolic calcium concentration, oxidative stress, inflammation, and uremic toxins. If erythrocyte synthesis does not compensate for the accelerated eryptosis, anemia may develop. Moreover, enhanced eryptosis contributes to the pathogenesis of different clinical diseases, such as diabetes, sepsis, metabolic syndrome, and uremia. In particular, in patients with chronic kidney disease (CKD), deficiencies of erythropoietin and iron may further reduce the lifespan of RBCs. In this review, we focused on eryptosis in CKD and end-stage renal disease on peritoneal dialysis (PD) and hemodialysis (HD).

## 1. Introduction

Erythrocytes, or red blood cells (RBCs), represent the most abundant cells of the vertebrate bloodstream (approximately 45% of human blood volume). In contrast to typical nucleated cells, mature RBCs are filled with hemoglobin and do not have either a nucleus or organelles. They are highly specialized in gas exchange, thanks to their flexible, biconcave shape. The biconcave shape of red blood cells increases their surface area-to-volume ratio, which optimizes the diffusion of oxygen and carbon dioxide across the cell membrane. This geometry also contributes to the cell’s remarkable flexibility, allowing RBCs to deform as they traverse narrow capillaries and splenic sinusoids without rupturing, thus ensuring efficient gas exchange even in the microcirculation. In particular, RBCs have evolved to efficiently transport oxygen and carbon dioxide throughout the body. They have an approximate size of 7–8 µm in diameter [1,2,3,4].

The membrane of RBCs consists of an intricate structure able to interact with a wide range of both external (xenobiotic) and internal (endogenous) factors. It is fundamental for the primary function of gas exchange, as well as for the adaptation to various environmental and physiological changes, without changes in cellular shape, flexibility, and overall functionality. The membrane’s sensitivity is critical for an appropriate response by RBCs to changes in oxygen levels, pH, and other factors, which are vital for body homeostasis. Moreover, RBCs must also interact with external substances, such as drugs or toxins, which may affect their function and survival. Therefore, their membrane represents not only a barrier but also a dynamic interface for complex interactions between the body’s internal and external environment [1,2,3,5,6,7].

The average lifespan of human RBCs is approximately 120 days, while mouse erythrocytes typically survive for approximately 40 days. During their life, RBCs are constantly transported throughout the body via the circulatory system, with a stable presence in different tissues and organs. As they circulate through the bloodstream, RBCs deliver vital oxygen to cells, supporting cellular respiration and energy production. In addition to oxygen transport, they also ensure carbon dioxide removal from tissues to the lungs for exhalation [1,2].

In adults, RBCs result from a process called erythropoiesis, which takes place in the bone marrow. During erythropoiesis, defective RBC maturation and overproduction are avoided. Indeed, different molecules regulate this process, ranging from cytokine signaling mechanisms responsible for extrinsic regulation of RBC production to intrinsic transcriptional pathways necessary for effective erythropoiesis.

The erythropoietic process starts with hematopoietic stem cells, progressing through stages including myeloid progenitors, megakaryocytic-erythroid progenitors, burst-forming unit-erythroid (BFU-e), colony-forming unit-erythroid (CFU-e), proerythroblasts, and various stages of erythroblasts (basophilic, polychromatic, and orthochromatic). Eventually, reticulocytes mature into fully functional RBCs. A critical part of this maturation process is the removal of the nucleus (enucleation) and organelles during the erythroblast and reticulocyte stages. This clearance is essential because it creates more space for hemoglobin, ensuring that the RBC maintains its biconcave shape, which is crucial for gas exchange. The process of enucleation is tightly controlled by various molecular factors, including transcription factors (e.g., FOXO3, E2F2), miRNAs (e.g., miR-30a, miR-191, miR-181a), cytoskeletal proteins (e.g., F-actin, dynein, tropomodulin), and kinases (e.g., p38 MAPK) [8,9,10]. Mitochondrial and organelle removal occurs through processes like macroautophagy or mitophagy. This process also involves the clearance of lysosomes, peroxisomes, Golgi apparatus, endoplasmic reticulum, and ribosomes in an autophagy-dependent manner [11,12,13].

RBCs lose their ribosomes progressively with aging, thus becoming incapable of synthesizing proteins [13]. Moreover, reduced metabolic activity, changes in cell morphology, decreased cell volume, and modifications in the cell surface are generally observed in mature RBCs [14]. Similarly, damaged RBCs undergo the same physiological modifications. Aged or damaged RBCs are cleared by the reticuloendothelial system (RES) located in the liver, spleen, and bone marrow. In particular, the liver is the main organ responsible for the clearance of aged and damaged RBCs, which are generally removed by the Kupffer cells (KCs) [15].

Even though erythroid precursor cells containing organelles may undergo different forms of regulated cell death, such as apoptosis, necroptosis, or ferroptosis, mature RBCs—which lack a nucleus and organelles—are unable to activate these classical pathways that typically require transcriptional activity and de novo protein synthesis. Nevertheless, mature RBCs can still undergo eryptosis, a distinct form of programmed cell death that is regulated through the modulation of pre-existing proteins and ion fluxes, particularly calcium influx. While mature RBCs cannot initiate new signaling cascades through protein expression, they retain components such as Fas, caspases, and RIPK1 inherited from earlier stages of erythropoiesis. These molecules, together with changes in intracellular calcium, oxidative stress, and energy depletion, mediate eryptosis through a post-translational control mechanism. Thus, eryptosis represents a simplified, but still regulated, form of cell death that is compatible with the limited biochemical capabilities of mature RBCs [16].

RBCs undergo programmed cell death called eryptosis, which is similar to apoptosis [5,6,7]. If erythrocyte synthesis does not compensate for the accelerated eryptosis, anemia may develop. Eryptosis is known to contribute to the pathogenesis of different clinical conditions, such as diabetes, sepsis, metabolic syndrome, and uremia [5,17,18,19,20,21,22].

**Figure 1:**
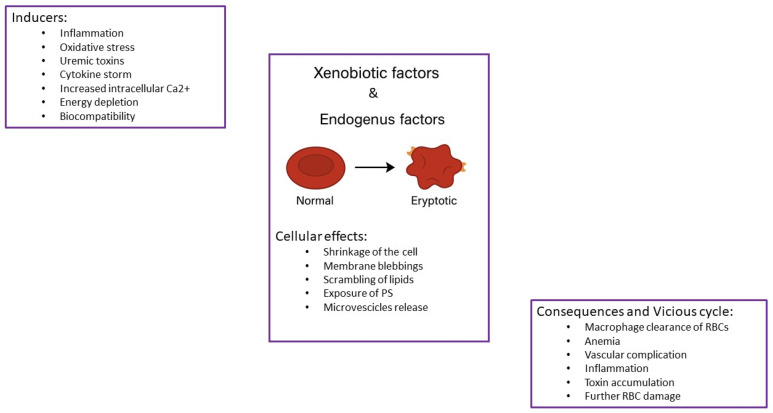
Mechanisms and inducing factors of eryptosis. Eryptosis is a programmed cell death of red blood cells characterized by cell shrinkage, membrane blebbing, and exposure of phosphatidylserine (PS) on the outer cell surface. These changes signal recognition and removal by macrophages. Key factors triggering eryptosis include increased cytosolic Ca^2+^ concentration, oxidative stress, inflammation, and the presence of uremic toxins.

Eryptosis is characterized by cell shrinkage, membrane bleb formation, and lipid scrambling, which all result in the exposure of the aminophospholipid phosphatidylserine (PS) on the outer surface of the RBCs. These cells thus become ready for removal. Indeed, they may bind to endothelial cells lining blood vessels, and this represents a signal for macrophages, which are able to engulf the altered RBCs. The removal of aged or damaged RBCs from the bloodstream is crucial for the maintenance of the health of the circulatory system [21,23,24]. Eryptosis is totally different from hemolysis. Hemolysis refers to the breakdown or rupture of red blood cells (RBCs), resulting in the release of hemoglobin and other intracellular components into the bloodstream. This can disrupt the balance of electrolytes and lead to complications such as jaundice, kidney damage, and anemia [25,26]. Among the factors able to induce eryptosis, increased cytosolic Ca^2+^ concentration, oxidative stress, inflammation, and several uremic toxins should be considered (Figure 1).

### 1.1. The Aim of This Study

In this review, we focused on the role of eryptosis in the progression of renal anemia in patients with chronic kidney disease (CKD). Moreover, we analyzed the effects of uremic toxins on erythrocyte lifespan in this population of patients. Finally, we investigated the possible effects of the two modalities of renal replacement therapy on eryptosis in patients with end-stage renal disease treated with peritoneal dialysis (PD) or hemodialysis (HD).

### 1.2. Literature Search Tools

A complete search in the PubMed and Cochrane databases was carried out with these search strings: (“eryptosis” OR “RBC apoptosis”) AND (“renal disease”), (“eryptosis” OR “RBC apoptosis”) AND (“CKD OR chronic kidney disease”), (“eryptosis” OR “RBC apoptosis”) AND (“dialysis”), (“eryptosis” OR “RBC apoptosis”) AND (“hemodialysis”), (“eryptosis” OR “RBC apoptosis”) AND (“peritoneal dialysis”).

Furthermore, PubMed was used to identify narrative or systematic reviews and published studies using specific terms to elaborate and improve our results. The references of the retrieved papers were used to identify additional relevant publications. The first-choice criteria for article selection were as follows: relevance of topic, evaluation of title and abstract, meta-analysis, clinical trial, original articles, guideline reports, systematic reviews, and recent papers.

## 2. Bibliographic Research Results

Table 1 reports results for the literature research for each search string (31 March 2025) (Table 1).

## 3. Chronic Kidney Disease Setting

In patients with chronic kidney disease (CKD), the development of anemia is a typical complication that is associated with impaired quality of life [27], increased risk for both cardiovascular events and hospitalizations [28,29], and cognitive decline [30]. In addition to the well-known factors involved in the pathogenesis of renal anemia, such as decreased erythropoietin production and iron deficiency, eryptosis is also known to contribute to the development of this clinical condition [31,32,33]. Moreover, in patients with CKD, oxidative stress, inflammation, energy depletion, and uremic toxins exacerbate eryptosis, thus worsening anemia [21,34,35]. All of these conditions worsen with the progression of renal damage, creating a vicious circle.

In chronic kidney disease, iron deficiency may be worsened by proteinuria, which may lead to urinary loss of transferrin-bound iron, particularly when proteinuria reaches the nephrotic range [36]. In a recent animal study, Bissinger et al. demonstrated a correlation between anemia and eryptosis in proteinuric kidney disease with severely impaired renal function [37]. The authors described the development of renal anemia in mice with proteinuric kidney disease induced by either the administration of doxorubicin or an inducible podocin deficiency [37]. In both experimental models, anemia progressed from day 10 to day 30, in spite of increased circulating erythropoietin levels. The authors reported an increased percentage of PS-exposing RBCs, as well as higher levels of reactive oxygen species and ceramide, thus suggesting an accelerated eryptosis [37]. Also, uremic toxins, which increase with the progression of renal impairment, induce both oxidative damage and cellular stress, thus leading to premature RBC death. Furthermore, the chronic inflammation typical of CKD exacerbates eryptosis through the production of pro-inflammatory cytokines responsible for the increase in intracellular calcium levels, a key trigger for the process. Given the critical role of eryptosis in the pathophysiology of renal anemia, targeting its underlying mechanisms could provide new therapeutic strategies to manage anemia in CKD patients [5,37,38,39,40].

In a recent in vitro study, Bonan et al. described a novel pathway in the pathogenesis of renal anemia through the examination of the cytotoxic effects of uremic plasma on both healthy RBCs and healthy CD14++/CD16+ monocytes. Serum from CKD patients was able to induce eryptosis in healthy RBCs, as well as a pro-inflammatory phenotype in monocytes [22]. This finding highlights the dual impact of uremic plasma on both RBCs and the immune system. The accelerated death of RBCs further contributes to the development of renal anemia in CKD patients. Moreover, the activation of pro-inflammatory monocytes plays a role in renal disease progression and other complications [22].

The role of uremic toxins in eryptosis, particularly in the context of CKD, has been widely investigated: indoxyl sulphate [41], acrolein [42], vanadate [43], indole-3-acetic acid [44], and urea and p-Cresol [45] may directly trigger eryptosis. Specifically, these uremic toxins have been found to induce high levels of eryptosis in patients with CKD. Indeed, the accumulation of these molecules due to impaired renal function leads to significant stress on RBCs, promoting their premature destruction.

Uremic toxins involved in eryptosis are mainly generated through the metabolism of amino acids and other nitrogenous compounds by the intestinal microbiota, followed by hepatic biotransformation and impaired renal clearance. For example, indoxyl sulfate is derived from dietary tryptophan. In the colon, the bacterial metabolism of tryptophan produces indole, which is absorbed and transported to the liver, where it is hydroxylated and sulfated by cytochrome P450 enzymes and sulfotransferases (SULTs) to form indoxyl sulfate. p-Cresol originates from the microbial breakdown of tyrosine and phenylalanine into p-hydroxyphenylacetic acid and, subsequently, into p-Cresol. In the liver, sulfotransferases convert it into p-cresyl sulfate, the main circulating form. Acrolein, a highly reactive α,β-unsaturated aldehyde, can be produced endogenously through lipid peroxidation, the metabolism of polyamines (via amine oxidases), and from the degradation of threonine. Acrolein can also form as a byproduct during oxidative stress and is poorly metabolized in CKD (Table 2). These compounds are normally excreted via the kidneys, but in CKD, impaired renal clearance leads to their accumulation in plasma. Their molecular reactivity (e.g., with proteins, lipids, and membranes) contributes directly to oxidative stress, inflammation, and eryptosis in RBCs.

Indoxyl sulfate has been demonstrated to induce erythrocyte shrinkage and cell membrane scrambling, both hallmarks of eryptosis. They presumably resulted from an increase in cytosolic calcium concentration, leading to an increased externalization of PS on the surface of cells. Additionally, the same study described the role of indoxyl sulfate in the enhancement of ceramide levels, which is a well-known factor contributing to eryptosis [41].

Dias et al. supported the role of indoxyl sulfate in the pathogenesis of renal anemia. They suggested that indoxyl sulfate could trigger oxidative stress and promote eryptosis via the Organic Anion Transporter 2, or OAT2, an NADPH oxidase activity-dependent and GSH-independent mechanism [46].

Accelerated eryptosis in end-stage renal disease could trigger thrombosis through the adhesion of PS-exposing erythrocytes to the vascular wall, a process that is expected to interfere with blood flow and stimulate blood clotting [24,47]. In a study by Gao et al., the uremic solutes indoxyl sulfate and indole-3-acetic acid were reported to induce a procoagulant phenotype in RBCs through PS exposure and microparticle release [44]. These mechanisms provide binding sites for factor Xa and prothrombinase complexes, thus promoting the coagulation cascade reaction with a dramatic increase in thrombin production [44].

Similarly, acrolein seems to stimulate oxidative stress, thus leading to elevated ceramide levels responsible for an increase in cytosolic calcium concentration and, consequently, for increased eryptosis levels [42]. Indeed, acrolein’s cytotoxic properties are related to the formation of acrolein-cysteine conjugates, contributing to oxidative stress and cellular apoptosis [48,49]. Moreover, acrolein forms adducts with guanine, adenine, and cytosine, thus resulting in DNA damage [50]. In 2024, Kopera et al. reported alterations in the structure of RBC membranes induced by acrolein due to changes in membrane composition, cytosolic proteins, and osmotic sensitivity. The extent of these changes was a dose-dependent effect according to the concentration of acrolein [51]. In particular, a decrease in lipid fluidity in the hydrophobic region of the lipid monolayer due to changes in protein–lipid interactions was observed. Furthermore, the authors demonstrated an increased mobility of the membrane cytoskeletal proteins, resulting in a higher osmotic sensitivity of erythrocytes [42]. A reduction in the total non-enzymatic antioxidant cellular potential was also found with a consequent increase in the level of reactive oxygen species [42].

Also, vanadate has been found to trigger eryptosis in subjects with CKD through the impairment of ATP production, with a consequent state of energy deficiency in RBCs. Additionally, vanadate is able to inhibit glycolysis within RBCs, further compromising their energy balance and functionality and potentially exacerbating the effects of eryptosis [43].

Table 3 summarizes molecular pathways and cellular mechanisms of eryptosis induced by key uremic toxins (Table 3).

All these findings about the effect of uremic toxins on eryptosis were validated through in vitro studies. In these experiments, RBCs from healthy individuals were exposed to different increasing concentrations of the uremic compounds at different time points. Also, eryptosis levels were evaluated at different time points and doses of exposure. Virzì et al. evaluated the percentage of eryptosis in healthy RBCs treated with variable concentrations of IL-6, IL-1β, urea, and p-Cresol (comparable to the plasma levels found in CKD patients) at different time points. The study investigated the cytotoxic effects of these substances on RBCs in an in vitro setting. The results supported the negative impact of both cytokines and uremic toxins on RBC viability, thus promoting eryptosis. The most significant cytotoxic effects, leading to the highest levels of eryptosis, were observed at higher concentrations and after longer exposure times (24 h), showing a time- and dose-dependent relationship (time- and dose-dependent effect) [45].

These in vitro findings were further supported by another study involving 25 CKD patients. Indeed, Clementi et al. reported higher eryptosis levels in patients with CKD (stage G4 and G5) compared to those in the early stages of renal damage (stage G1, G2, and G3). Additionally, the authors found a strong relationship between oxidative stress, inflammation, uremic toxins, and eryptosis. It is likely that in CKD patients, uremic toxins and reactive oxygen species (ROS) promote inflammation and oxidative stress by stimulating polymorphonuclear lymphocytes and inducing the release of inflammatory cytokines. All of these contributing factors collectively result in significant damage to the structure of the RBC membranes in patients with CKD, with consequent decreased survival [52].

### End-Stage Renal Disease: Hemodialysis (HD) and Peritoneal Dialysis (PD)

Abed et al. investigated the potential impact of end-stage renal disease (ESRD) on eryptosis, reporting significantly higher levels of RBC death in patients undergoing HD compared to healthy individuals. This research highlighted the association between ESRD and eryptosis, underlying the importance of understanding how ESRD may influence RBC survival, and the potential role of eryptosis in anemia in patients receiving HD [53].

Several studies have examined eryptosis levels before and after (pre- and post-) hemodialysis sessions [53,54,55]. Unfortunately, inconsistent results were obtained. One possible reason for the lack of consistency is the small sample size of the study populations of these investigations, which may not have provided sufficient statistical power to detect clear trends. Moreover, different studies analyzed only a single HD session or a very limited number of sessions, which may not accurately reflect the overall effects of HD on eryptosis over time. The variability in study design and methodology may have further contributed to the differing outcomes, highlighting the need for larger, more comprehensive studies to better understand the relationship between hemodialysis and eryptosis.

In an interesting study on HD patients, Hefny et al. explored the connection between parathyroid hormone (PTH) and phosphorus and eryptosis in 85 patients with stage 5 dialysis-dependent chronic kidney disease. Through linear regression analysis, they demonstrated that PTH levels were independently associated with the percentage of eryptosis, as assessed by flow cytometry. Based on these findings, hyperparathyroidism could worsen renal anemia in patients undergoing HD by contributing to eryptosis.

Recently, Marcello et al. used eryptosis as a marker to assess biocompatibility in the context of HD. Specifically, they investigated the safety of dialysis combined with hemadsorption (HA+HD) using the HA130 cartridge (Jafron Biomedical, Zhuhai City, China) in terms of biocompatibility and its efficacy in removing middle-molecule uremic toxins and protein-bound uremic toxins (PBUTs). They conducted a preliminary pilot analysis of an observational study focusing on seven chronic dialysis patients from their dialysis center, evaluating four dialysis sessions. The patients were treated with HA+HD using the HA130 cartridge (Jafron) during the early-week dialysis sessions, after which they returned to their usual dialysis prescription. No significant differences in eryptosis levels were found before and after the treatment. In this preliminary analysis, the authors demonstrated an efficient removal of middle-molecular-weight uremic toxins without compromising biocompatibility with the combination of dialysis and hemadsorption [56].

Furthermore, Bissinger et al. confirmed an elevated percentage of eryptotic RBCs in patients undergoing HD, with a positive correlation between the percentage of eryptotic RBCs and the levels of reactive oxygen species (ROS) and ceramide concentrations. They also reported a relationship between the percentage of PS-exposing erythrocytes and both the dosage of erythropoietin and the percentage of reticulocytes in these patients [57].

Erythropoietin (EPO), beyond its role in erythropoiesis, exerts cytoprotective effects on mature erythrocytes by modulating oxidative stress and apoptotic pathways. EPO has been shown to reduce eryptosis by attenuating calcium influx, reactive oxygen species (ROS) generation, and caspase activation. However, in ESRD, a subset of patients develops resistance to EPO therapy, often associated with chronic inflammation, iron dysregulation, and uremic toxin accumulation. These factors can compromise EPO receptor signaling, diminishing its protective effects on RBCs and potentially exacerbating eryptosis. Understanding the interplay between EPO responsiveness and RBC homeostasis may offer novel insights for optimizing anemia management in ESRD.

Moreover, Bissinger et al. compared eryptosis levels between patients on peritoneal dialysis (PD) and those on HD, revealing that eryptosis levels were higher in the PD group. In particular, in the PD subpopulation, a correlation between eryptosis levels and the dialysate volume was reported. The authors hypothesized that components of the dialysate, particularly those based on glucose, might contribute to the stimulation of eryptosis, suggesting a potential role of the dialysate composition in promoting RBC damage in this population of patients [57].

In contrast, Vos et al. reported comparable RBC survival rates in patients undergoing HD and PD in their study, performed on 14 HD patients and 5 PD patients. Using a different laboratory technique, specifically the chromium-51 labeling method, to track RBC survival, no significant difference in RBC longevity between the two groups was reported [58]. The results are in contrast with previous findings supporting a marked difference in eryptosis between HD and PD patients, underlying the need for further studies to better understand the factors influencing RBC survival in these populations of patients [57]. Generally, the sample size of peritoneal dialysis (PD) patients included in studies is relatively small, making it difficult to draw definitive conclusions about the specific mechanisms underlying eryptosis in this study population. The limited number of PD patients studied hinders the ability to fully understand the pathogenesis of eryptosis in these individuals, and results may not be representative of the broader PD patient population [53,57,59,60].

In the study of Meyring et al., the relationship between erythrocyte sodium sensitivity and eryptosis was investigated in patients with chronic kidney disease undergoing hemodialysis and compared to that in healthy controls [54]. Compared to the control group, erythrocyte sodium sensitivity was higher in patients undergoing hemodialysis pre-treatment and did not change during the session. In contrast, eryptosis decreased during the hemodialytic treatment, but it was not correlated to erythrocyte sodium sensitivity. Therefore, the reduction in eryptosis post-treatment may be due to the removal of uremic toxins, even though it is likely multifactorial, while the relationship between eryptosis and erythrocyte sodium sensitivity needs further research.

Different dialysis modalities could distinctly influence RBC physiology. Conventional HD, while effective in removing small solutes, may induce mechanical trauma and oxidative stress on RBCs due to rapid fluid and solute shifts, use of bioincompatible membranes, and intermittent exposure to uremic toxins. In contrast, alternative modalities such as hemodiafiltration (HDF) and peritoneal dialysis (PD) offer more gradual and potentially biocompatible toxin removal, which may reduce erythrocyte injury and promote better RBC survival. However, despite these theoretical advantages, precise and comprehensive studies directly comparing the impact of different dialysis modalities on eryptosis and related parameters are still limited. Further well-designed clinical and mechanistic studies are needed to elucidate how specific dialysis strategies influence RBC physiology and to determine whether optimizing dialysis modality can reduce erythrocyte injury and improve anemia management in chronic kidney disease patients.

Over the past decade, there has been a significant increase in research focused on this topic. A growing number of studies have been performed to better understand the mechanisms involved in eryptosis in PD patients. Specifically, Virzì et al. confirmed that the rate of eryptosis is significantly higher in stable peritoneal dialysis (PD) patients compared to that in healthy control subjects. The study, conducted on 46 PD patients, also investigated the possible effects of different comorbidities, such as diabetes, hypertension, cardiovascular disease, and key PD parameters, on eryptosis levels. All these factors, including whether the patient was on continuous ambulatory or automated PD, Kt/V urea values (whether ≤1.7 or >1.7), and a history of peritonitis, did not seem to influence the rate of eryptosis. In contrast, significantly lower levels of eryptosis in PD patients with a weekly creatinine clearance of ≥45 L/week/1.73 m^2^, as well as in those with residual diuresis (n = 23), were reported. In the PD patients with residual diuresis, significant negative correlations were observed between the percentage of eryptosis and both residual glomerular filtration rate (rGFR) and diuresis volume. It may be speculated that the levels of eryptosis increase with the progressive loss of residual diuresis and rGFR, and it is likely due to a decrease in the clearance of uremic toxins. Therefore, as renal function declines, the accumulation of uremic toxins progresses, thus further promoting RBC damage and death in this population of patients [60].

To better understand the association between eryptosis and inflammation in PD patients, the same authors investigated eryptosis in the context of PD-related peritonitis, which is one of the most common and serious complications in this population of patients. Firstly, they compared eryptosis levels and systemic inflammatory markers (such as CRP, IL-6, and IL-1β) in 31 PD patients with acute peritonitis and 34 PD patients with no history of systemic inflammation or peritonitis in the past three months, representing the control group. On the first day of peritonitis, the percentage of eryptosis was three times higher in the PD patients with peritonitis compared to the control group. Furthermore, strong positive correlations were reported between all the inflammatory indices (CRP, IL-6, IL-1β) and eryptosis levels. These data suggest the important role of the inflammatory response triggered by peritonitis in the increase in eryptosis levels in PD patients [61]. Supporting this point, Bester et al. conducted a study aimed at investigating the impact of IL-1β, IL-6, and IL-8 on the structure of RBCs and platelets. They demonstrated that all three interleukins promote hypercoagulability and alterations in RBC membranes. Specifically, IL-8 had a notable effect on erythrocyte structure, leading to visible changes in the cell membrane and the initiation of eryptosis [62]. Additionally, C-reactive protein (CRP) has been identified as a trigger for eryptosis, with a strong association between CRP levels and eryptosis rate in acute inflammatory conditions, such as peritonitis and acute appendicitis [61,63]. These findings were further supported by in vitro experimental data, which evaluated the cytotoxic effects on healthy red blood cells (RBCs). The studies found that elevated CRP levels in inflammatory states significantly contribute to the induction of eryptosis, emphasizing the role of CRP in mediating RBC damage [61,62,63].

Finally, based on previous findings, Virzì et al. investigated the relationship between systemic eryptosis and specific biomarkers of peritonitis in the PD-effluent (PDE), including pWBC (peritoneal white blood cells), pNGAL (peritoneal Neutrophil Gelatinase-Associated Lipocalin), and the inflammatory cytokines IL-6 and IL-1β. These data demonstrated a significant positive correlation between the levels of eryptosis and all the examined peritoneal biomarkers of peritonitis, thus highlighting the connection between the systemic effects of eryptosis and local inflammation occurring in the peritoneum of PD-related peritonitis patients. The study also supported the concept that eryptosis is primarily influenced by the composition of the blood, particularly in inflammatory conditions [64]. Interestingly, the study reported that the degree of eryptosis observed on the first day of peritonitis did not correlate directly with the patient’s prognosis, suggesting that eryptosis levels at the onset of peritonitis do not represent a reliable predictor of clinical outcomes in these patients [64]. Additionally, the induction of eryptosis by peritonitis was confirmed in an in vitro model. In this model, healthy RBCs from donors were exposed to the plasma of PD patients with peritonitis, with a consequent increase in eryptosis levels. This result further supports the idea that the inflammatory environment associated with peritonitis contributes directly to the induction of eryptosis, suggesting that both local and systemic inflammation play a role in RBC damage in PD patients [64].

## 4. Translational and Clinical Perspectives

While most of the current evidence regarding eryptosis in renal disease arises from in vitro or observational studies, its potential clinical applications are increasingly being recognized. One relevant example is peritoneal dialysis-related peritonitis, where eryptosis levels have been shown to increase significantly in response to inflammation. In these patients, eryptosis positively correlates with both systemic (e.g., CRP, IL-6, IL-1β) and local (e.g., pNGAL, peritoneal WBC) markers of inflammation. This suggests that eryptosis may reflect the inflammatory burden and could help identify patients at higher risk of persistent or inflammation-driven anemia.

Given that post-peritonitis anemia is a frequent and clinically relevant issue in peritoneal dialysis patients, measuring eryptosis levels during and after inflammatory episodes might offer useful diagnostic insights and support clinical decision-making. Such an approach could help clinicians better differentiate between anemia due to reduced erythropoietin production, iron deficiency, or increased erythrocyte destruction.

An improved understanding of RBC dysfunction in ESRD has important clinical implications. Recent studies have highlighted that both disease progression and therapeutic interventions, such as erythropoietin (EPO) administration and dialysis modality, significantly affect RBC physiology and survival. For instance, Georgatzakou et al. demonstrated that RBCs from EPO non-responsive ESRD patients show more severe morphological alterations, increased membrane vesiculation, and dysregulated removal signaling compared to responders. These changes were accompanied by a reduced expression of key membrane regulators such as CD47 and CD59, potentially contributing to premature erythrocyte clearance and anemia [65]. Moreover, the dialysis modality itself may influence RBC homeostasis. Hemodiafiltration (HDF), though associated with improved pre-dialysis redox balance and reduced vesiculation compared to conventional hemodialysis (HD), was shown to transiently exacerbate post-dialysis eryptosis and oxidative stress, possibly due to the clearance of circulating antioxidants like uric acid [66,67]. This suggests a nuanced trade-off in choosing dialysis strategies and supports the potential utility of adjunctive antioxidant therapy during HDF. Proteomic analyses have further revealed that prolonged exposure to the uremic environment induces extensive remodeling of RBC proteins, which may correlate with cardiovascular risk and mortality. Alterations in intracellular hemoglobin content, glucose transporter expression, and redox-sensitive membrane proteins indicate that RBCs adapt to chronic renal stress in ways that may be clinically relevant [68,69,70,71]. These findings support the investigation of eryptosis-related markers (e.g., phosphatidylserine exposure, intracellular Ca^2+^, calpain activation) as potential diagnostic or prognostic biomarkers and as targets for personalized anemia management in ESRD.

From a practical standpoint, eryptosis could also serve as a useful biomarker to guide the management of anemia and to monitor the overall burden of uremic toxins, particularly in CKD patients who are not yet receiving dialysis. In this population, the early detection of enhanced eryptosis might allow for timely therapeutic interventions aimed at reducing oxidative stress and systemic inflammation, thus potentially slowing disease progression and improving quality of life.

Although further prospective studies are required to validate eryptosis as a biomarker or therapeutic target, its inclusion in clinical monitoring protocols—especially in inflammatory or pre-dialytic settings—may represent a promising avenue for the personalized management of anemia and uremic toxicity in CKD.

## 5. Conclusions

In conclusion, this review focused on eryptosis in chronic kidney disease and emphasized its strong connection with inflammation and oxidative stress. While our understanding of the exact mechanisms through which eryptosis contributes to renal disease progression remains incomplete, existing evidence supports its significant involvement in the pathophysiology of kidney disease. It is evident that further research is essential to explore eryptosis more deeply, particularly its potential as a key pathogenic factor in renal dysfunction and damage. Additionally, recent studies suggest that eryptosis may be a valuable marker for evaluating biocompatibility in dialysis and other extracorporeal therapies, helping clinicians in the assessment of the safety and efficacy of such treatments. Given the increasing use of extracorporeal therapies in managing kidney disease, understanding the role of eryptosis in these contexts could have important clinical implications for improving patient outcomes and optimizing therapeutic interventions.

## Figures and Tables

**Table 1:** The literature search results for each search string (31 March 2025).

	Search String	Papers
Eryptosis OR RBC apoptosis	Renal disease	48
	CKD	16
	Dialysis	18
	HD	17
	PD	6

**Table 2:** Biochemical origin and mechanisms of indoxyl sulfate, p-Cresol, and acrolein involved in eryptosis.

Uremic Toxin	Precursor(s)	Site of Production	Enzymes Involved	Mechanism of Action in Eryptosis
Indoxyl sulfate	Tryptophan	Colon → Liver	Bacterial tryptophanase, cytochrome P450, SULTs	Increases ROS and intracellular Ca^2+^; promotes PS exposure and cell shrinkage
p-Cresyl sulfate	Tyrosine, Phenylalanine	Colon → Liver	Bacterial fermentation, sulfotransferases	Induces oxidative stress; enhances membrane damage and eryptosis signaling
Acrolein	Lipids, Polyamines, Threonine	Endogenous (various tissues)	Amine oxidases, lipid peroxidation pathways	Alters membrane structure; increases Ca^2+^ and ceramide; triggers PS externalization

**Table 3:** Molecular pathways and mechanisms of eryptosis induced by key uremic toxins.

Uremic Toxin	Molecular Pathways Activated	Mechanism Summary in Eryptosis
Indoxyl Sulfate	- Activation of NADPH oxidase - Uptake via Organic Anion Transporter 2 (OAT2) - ROS generation	Increases oxidative stress and intracellular Ca^2+^, promoting membrane PS exposure.
Acrolein	- Lipid membrane modification - Oxidative damage to cytosolic and membrane proteins	Alters membrane fluidity and integrity, triggering eryptosis signaling.
Vanadate	- Inhibition of glycolysis - Impairment of ATP production—Energy depletion	Induces eryptosis by causing energy failure in RBCs.
p-Cresol	- ROS production - Oxidative stress	Promotes Ca^2+^ influx and phosphatidylserine exposure on the erythrocyte surface.
Homocysteine	- Oxidative stress - Glutathione depletion	Weakens antioxidant defenses, sensitizing cells to eryptosis.
Methylglyoxal	- Glycation of membrane proteins - Oxidative stress	Causes membrane dysfunction and promotes eryptotic signaling.

## Data Availability

No new data were created or analyzed in this study.
